# VGLL-fused intraparenchymal schwannoma with* EWSR1*::*VGLL1* fusion: integrated clinicopathological and molecular characterization

**DOI:** 10.1186/s40478-026-02355-2

**Published:** 2026-06-25

**Authors:** Ryosuke Ikemachi, Yohei Inoue, Yoshihiro Otani, Kyohei Kin, Ryo Mizuta, Yasuhiko Hattori, Kohei Izumihara, Shuichiro Hirano, Joji Ishida, Fumiyo Higaki, Kyosuke Horikawa, Kotaro Yoshio, Takahiro Tsuchiya, Satoru Miyawaki, Koichi Ichimura, Sumihito Nobusawa, Shota Tanaka

**Affiliations:** 1 Department of Neurological Surgery, Okayama University Graduate School of Medicine, Dentistry and Pharmaceutical Sciences, 2-5-1 Shikata-cho, Kita-ku, Okayama-City, Okayama 700-8558 Japan; 2 Department of Pathology, Kyorin University Faculty of Medicine, 6-20-2 Shinkawa, Mitaka, Tokyo 181-8611 Japan; 3 Department of Neurosurgery, Okayama City General Medical Center, Okayama City Hospital, 3-20-1 Kitanagase Omote-machi, Kita-ku, Okayama-City, Okayama 700-0962 Japan; 4 Department of Radiology, Medical Development Field, Okayama University, 2-5-1 Shikata-cho, Kita-ku, Okayama-City, Okayama 700-8558 Japan; 5 Department of Pathology and Oncology, Okayama University Graduate School of Medicine, Dentistry, and Pharmaceutical Sciences, 2-5-1 Shikata-cho, Kita-ku, Okayama-City, Okayama 700-8558 Japan; 6 Department of Proton Beam Therapy, Okayama University Graduate School of Medicine, Dentistry, and Pharmaceutical Sciences, 2-5-1 Shikata-cho, Kita-ku, Okayama-City, Okayama 700-8558 Japan; 7 Department of Neurosurgery, Graduate School of Medicine, The University of Tokyo, 7-3-1 Hongo, Bunkyo-ku, Tokyo 113-8655 Japan; 8 Department of Human Pathology, Gunma University Graduate School of Medicine, 3-39-15 Showa-machi, Maebashi, Gunma 371-8511 Japan

**Keywords:** Intraparenchymal schwannoma, VGLL-fused schwannoma, *EWSR1*::*VGLL1* fusion, *VGLL1* mRNA expression

## Abstract

Intraparenchymal schwannomas are rare intracranial tumors confined to the brain parenchyma without connection to peripheral nerves. While conventional schwannomas are often associated with *NF2* inactivation, the pathogenesis of intraparenchymal schwannomas remains poorly understood. Recent molecular studies have identified a distinct subgroup characterized by VGLL family fusions, suggesting a unique pathogenetic mechanism. Here, we report a 24-year-old woman presenting with dizziness and facial palsy who underwent resection of a brainstem tumor that showed histopathological features consistent with schwannoma. DNA methylation profiling classified the tumor as a VGLL-fused schwannoma, and RNA sequencing identified an *EWSR1*::*VGLL1* fusion with markedly higher *VGLL1* mRNA expression compared to a reference cohort of 17 vestibular schwannomas. Copy number analysis revealed loss of chromosome 19 without loss of chromosome 22q. These findings support a potential role for *VGLL1* fusion in the pathogenesis of this rare tumor subtype. Importantly, this case further substantiates the emerging molecular subset of VGLL-fused intraparenchymal schwannomas and underscores the value of integrated molecular and histopathological analyses in the classification of central nervous system tumors.

## Introduction

Schwannomas are typically benign tumors originating from Schwann cells, which form the myelin sheath around peripheral nerves. They account for approximately 8% of all primary intracranial tumors [23], and the vast majority of cases arise from the eighth cranial nerve, commonly called vestibular schwannomas. In contrast, intraparenchymal schwannomas are extremely rare, accounting for less than 1% of all intracranial schwannomas [9, 19, 31]. This rare tumor displays the histological characteristics of a schwannoma and demonstrates immunohistochemical evidence of Schwann cell differentiation, yet it lacks any connection to peripheral nerves and is confined to the brain parenchyma.

Recent advancements in molecular diagnostics have begun to shed light on this rare entity. While peripheral schwannomas are frequently driven by *NF2* inactivation, additional driver alterations such as *SOX10* indel mutations and *SH3PXD2A*::*HTRA1* fusions have also been reported [1, 30, 33]. Notably, these alterations have not been reported in intraparenchymal schwannomas [10, 11]. Instead, a distinct DNA methylation profile has been identified in intraparenchymal schwannomas, along with recurrent VGLL family gene fusions, such as *CHD7*::*VGLL3* and *EWSR1*::*VGLL1* [25].

Here, we report a case of a VGLL-fused intraparenchymal schwannoma with an *EWSR1*::*VGLL1* fusion in a young adult woman. This case provides further clinicopathological and molecular insights into the spectrum of intraparenchymal schwannomas and suggests that VGLL family gene alterations may play a role in the pathogenesis of these tumors.

## Case presentation

A 24-year-old woman had been experiencing dizziness for a month, after which she developed facial palsy and visited an otolaryngologist. A peripheral facial nerve palsy was initially suspected, and she was treated with prednisolone. However, because her symptoms did not improve, magnetic resonance imaging (MRI) was performed. It revealed a tumor in the posterior fossa, and she was subsequently referred to our hospital. At the initial presentation, right abducens nerve palsy was also observed.

**Fig. 1:**
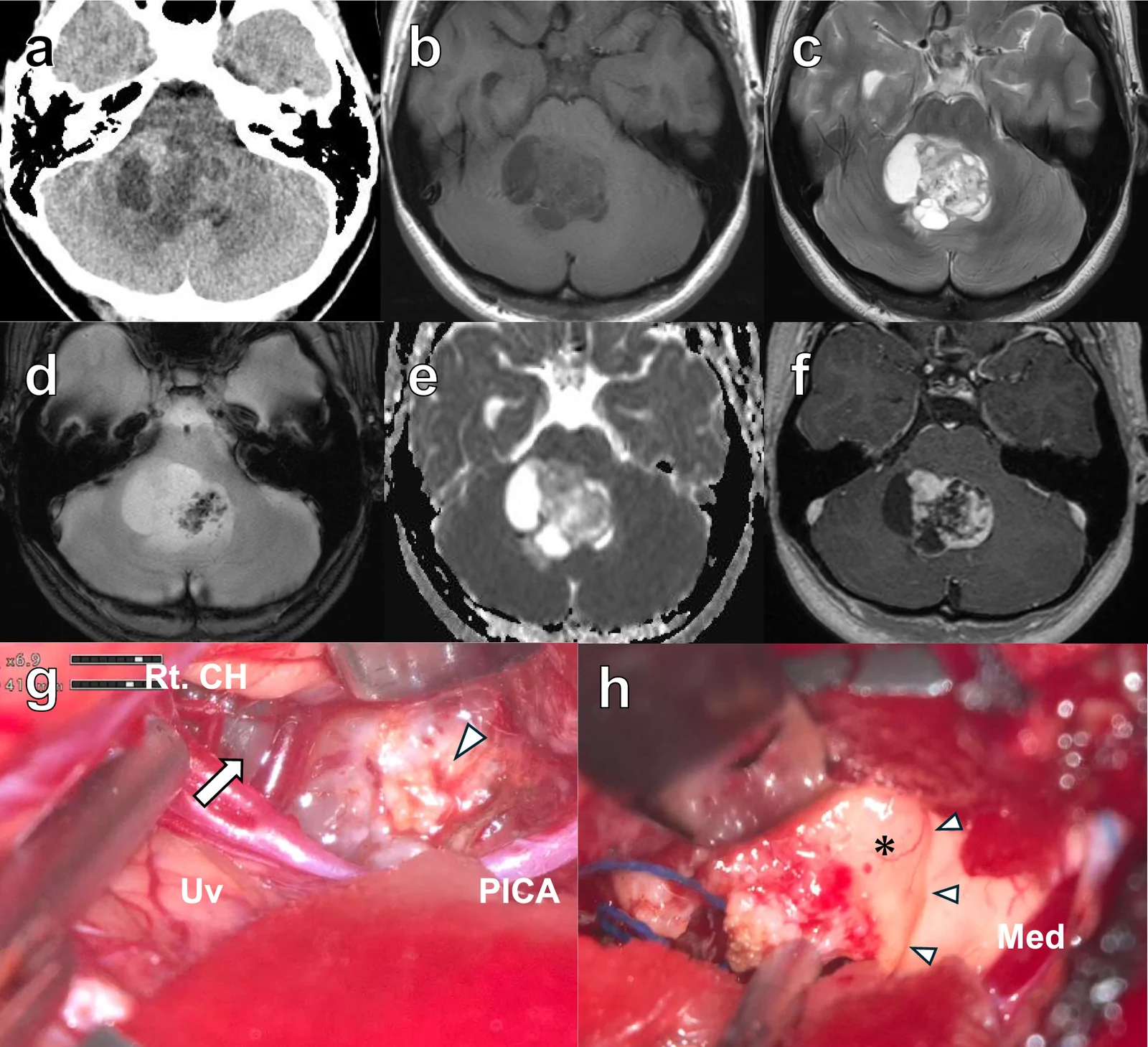
Radiological and intraoperative findings. Computed tomography (CT) and magnetic resonance imaging (MRI) findings. **a**–**g** CT and MRI revealed a tumor comprising mixed cystic and solid components; **a** non-contrast CT image, **b** T1-weighted MRI, **c** T2-weighted MRI, **d** T2^*^-weighted MRI, **e** ADC map, and **f** gadolinium-enhanced T1-weighted MRI. Intraoperative findings. **g** Upon opening the cerebellomedullary fissure, a tumor with both solid (arrowhead) and cystic (arrow) components were exposed. **h** The boundary (arrowhead) between the brainstem and the tumor (asterisk) was poorly demarcated, with strong adhesion. CT; Computed tomography, MRI; magnetic resonance imaging, Rt; right, CH; cerebellar hemisphere, Uv; uvula, PICA; posterior inferior cerebellar artery, Med; medulla

A head computed tomography (CT) scan revealed a tumor comprising mixed cystic and solid components, and no calcification was identified (Fig. 1a). MRI also showed a cystic and solid tumor. The solid component demonstrated hypointensity on T1-weighted images (T1WI) and hyperintensity on T2-weighted images (T2WI) (Fig. 1b and c). T2*-weighted images (T2*WI) revealed chronic hemorrhage (Fig. 1d). The apparent diffusion coefficient (ADC) value of the solid component suggested low cellularity (Fig. 1e). Heterogeneous enhancement was observed on gadolinium-enhanced MRI (Fig. 1f). The fourth ventricle was markedly displaced to the left by the tumor, without evidence of hydrocephalus, suggesting that the lesion originated within the cerebellar parenchyma. Based on these findings, the preoperative diagnosis was pilocytic astrocytoma, with ependymoma and medulloblastoma as differential diagnoses.

Surgery was performed via a midline suboccipital craniotomy. Upon opening the cerebellomedullary fissure, the tumor was exposed, consisting of solid and cystic components (Fig. 1g). The tumor was relatively soft, and internal decompression was performed using an ultrasonic aspirator. Intraoperative histopathological examination suggested a low-grade glioma. The frozen section revealed a cellular proliferation of tumor cells with small round to short spindle-shaped nuclei, focal nuclear palisading, and eosinophilic structures resembling Rosenthal fibers, without necrosis or conspicuous mitotic activity. Taken together with radiological findings, a low-grade glioma such as pilocytic astrocytoma was considered in the differential diagnosis. However, a definitive histological diagnosis could not be made intraoperatively. The boundary between the tumor and the cerebellar hemisphere was well defined, allowing careful dissection. In contrast, the boundary between the brainstem and the tumor was poorly demarcated, and the tumor was tightly adherent in this region (Fig. 1h). Therefore, this portion was partially left behind. Intraoperative findings suggested a brainstem origin.

**Fig. 2:**
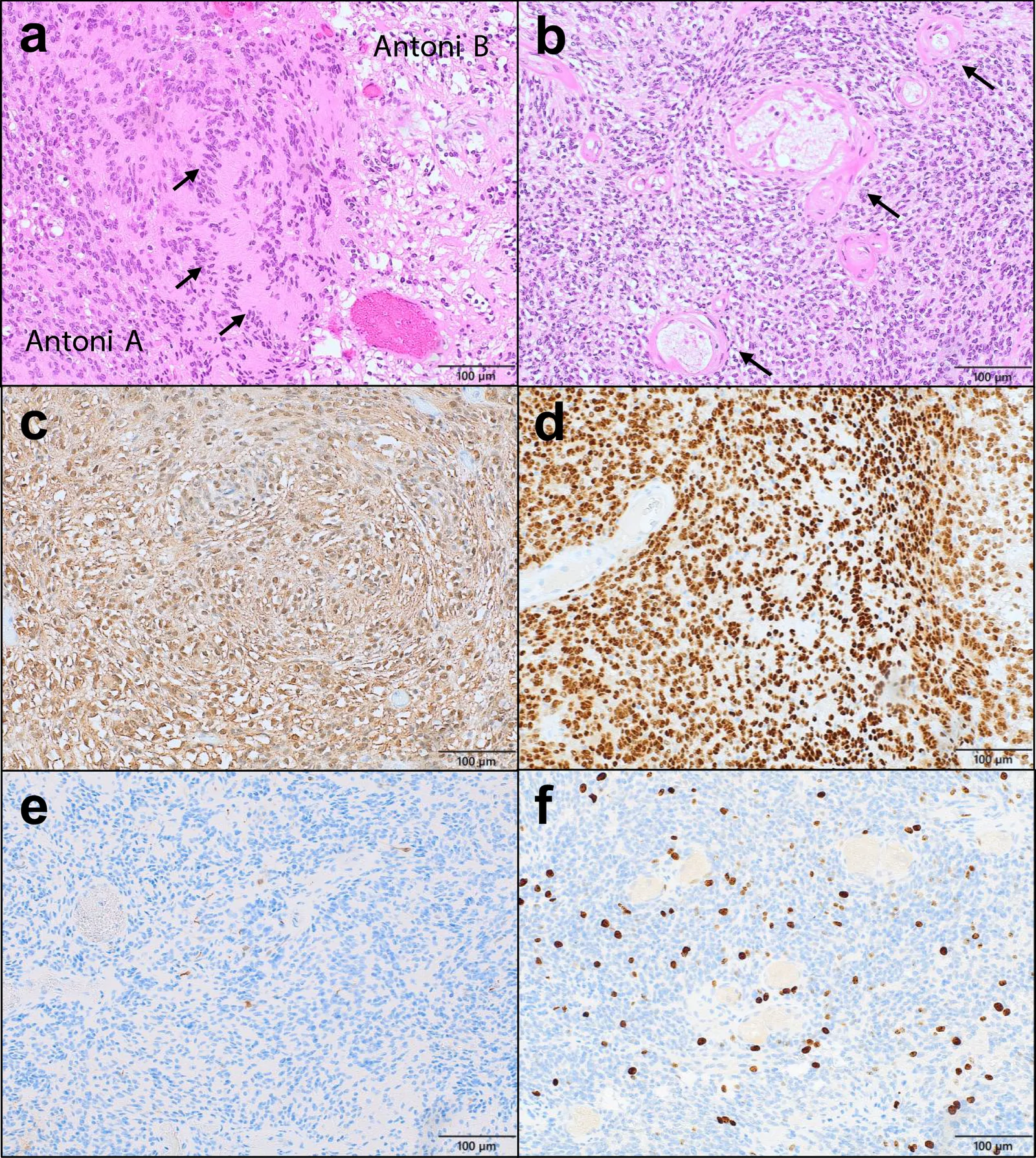
Histopathological findings. **a**, **b** Hematoxylin–eosin staining. **a** The tumor showed a biphasic architecture composed of hypercellular Antoni A areas with a fascicular pattern and hypocellular Antoni B areas with a loose, reticular pattern. Focal nuclear palisading and Verocay bodies were observed (arrows). **b** Hyalinized blood vessels were also observed (arrows). **c**–**f** Immunohistochemistry. Tumor cells showed positive staining for S-100 protein (**c**) and SOX10 (**d**), whereas GFAP staining was negative (**e**). The Ki-67 labeling index was 3.9% (**f**). Scale bars, 100 μm

Histopathological examination revealed a biphasic architecture composed of hypercellular Antoni A areas with a fascicular pattern and hypocellular Antoni B areas with a loose, reticular pattern. The tumor cells had round to short spindle-shaped nuclei. Focal nuclear palisading and Verocay bodies were observed (Fig. 2a). In addition, hyalinized blood vessels, necrosis, hemorrhage, and hemosiderin deposits were also observed (Fig. 2b). On immunohistochemistry, S-100 protein and SOX10 were positive (Fig. 2c and d). Stains for GFAP (Fig. 2e), OLIG2, synaptophysin, chromogranin A, neurofilament, EMA, D2-40, and BRAF V600E were negative. The Ki-67 labeling index was 3.9% (Fig. 2f). Histological analysis suggested intraparenchymal schwannoma.

**Fig. 3:**
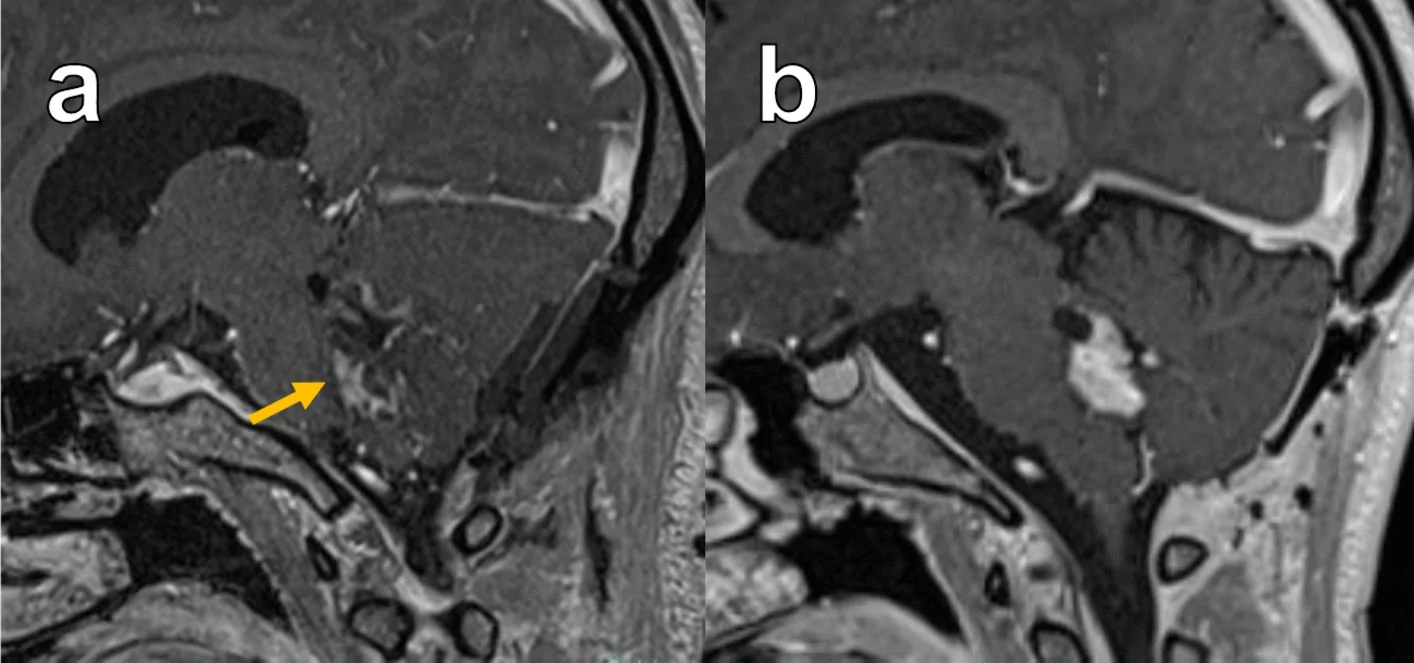
Postoperative course of radiological findings. **a** Postoperative MRI demonstrated near-total resection, with a small residual tumor adherent to the brainstem (arrow). **b** MRI at 5 months postoperatively showed slight enlargement of the residual tumor

Postoperative MRI demonstrated near-total resection of the tumor (Fig. 3a). Follow-up MRI at 5 months postoperatively showed slight enlargement of the residual tumor (Fig. 3b), and the patient subsequently underwent stereotactic radiotherapy (25 Gy in 5 fractions). MRI performed 6 months after radiotherapy demonstrated no evidence of tumor progression.

## Molecular analyses

### DNA methylation profile and copy number analysis

To further characterize the tumor, genome-wide DNA methylation profiling was performed using formalin-fixed paraffin-embedded (FFPE) tissue. Genomic DNA was extracted using the AllPrep DNA/RNA FFPE Kit (QIAGEN, Hilden, Germany) according to the manufacturer’s instructions. A total of 500 ng of genomic DNA was subjected to bisulfite conversion using the EZ DNA Methylation Kit (Zymo Research Corp., Irvine, CA, USA).

Bisulfite-converted DNA was hybridized to the Infinium MethylationEPIC v2.0 BeadChip (Illumina, San Diego, CA, USA) and scanned on an Illumina iScan System. Raw IDAT files were uploaded to the Heidelberg CNS Tumor Methylation Classifier (version 12.8) (https://app.epignostix.com/) and the NCI/Bethesda (Methylscape) classifier (version 2.0) (https://methylscape.ccr.cancer.gov/) to obtain DNA methylation–based tumor classification and copy number profiles [2].

**Fig. 4:**
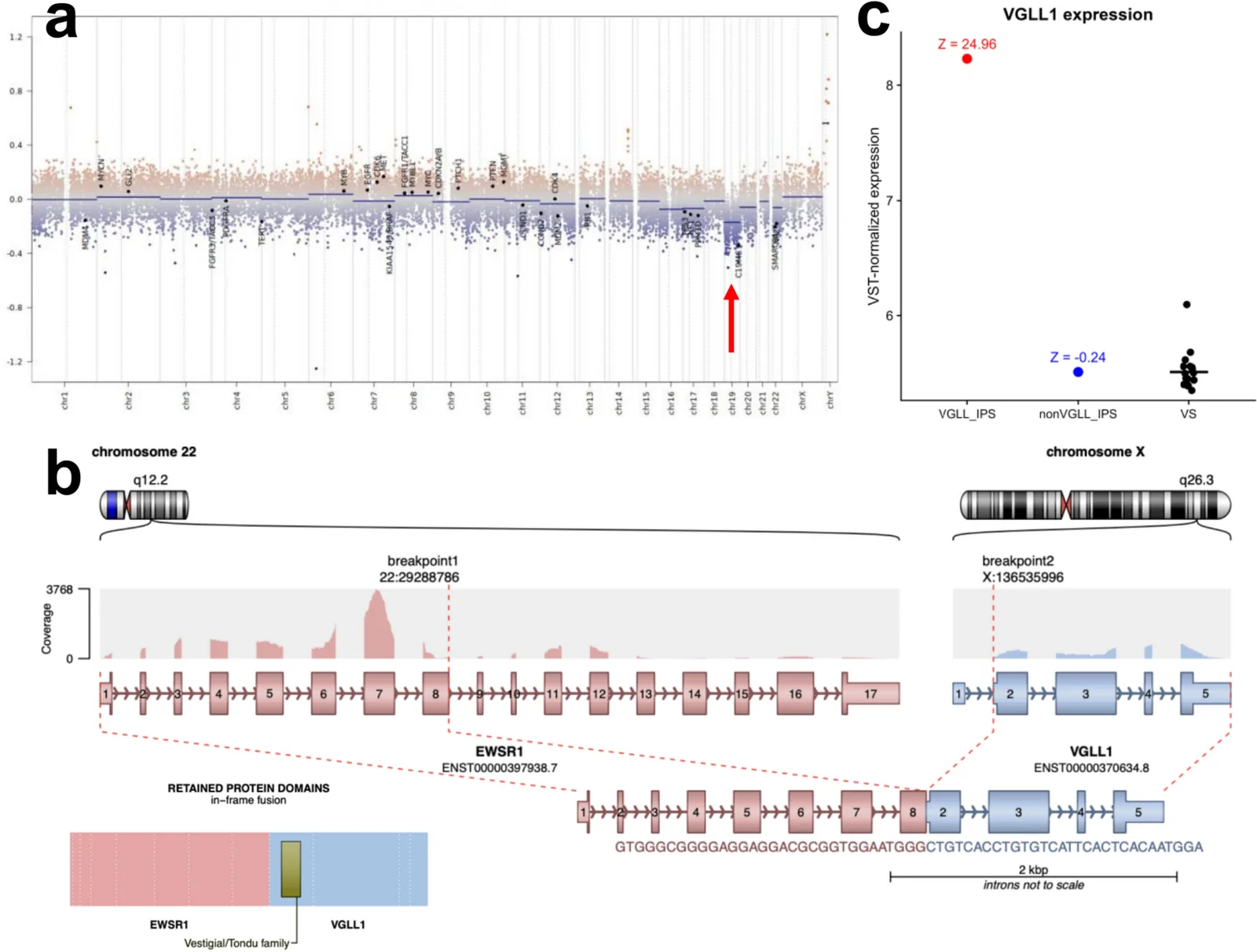
Molecular findings. **a** Copy number alteration analysis of chromosomes showed loss of chromosome 19. **b** Fusion gene analysis using Arriba identified an *EWSR1*::*VGLL1* gene fusion. **c** VST-normalized *VGLL1* mRNA expression in the present *EWSR1*::*VGLL1* fusion-positive intraparenchymal schwannoma (VGLL_IPS, red), a non-VGLL-fused intraparenchymal schwannoma from our previously reported cohort (nonVGLL_IPS, blue), and a vestibular schwannoma reference cohort (VS, n = 17, black). Each dot represents an individual sample. The Z-score indicate the deviation of the IPS sample relative to the VS reference cohort. *VGLL1* expression was markedly elevated only in the present case. VST; variance stabilizing transformation, IPS; intraparenchymal schwannoma, VS; vestibular schwannoma

In the Heidelberg CNS Tumor Methylation Classifier, the tumor was classified as “CNS schwannoma, VGLL-fused” with a calibrated score of 0.99998. Copy number alterations (CNAs) derived from methylation array data demonstrated loss of chromosome 19 without loss of chromosome 22q (Fig. 4a). The NCI/Bethesda (Methylscape) classifier yielded a concordant result, classifying the tumor as “CNS schwannoma, VGLL-fused” with a class mean score of 0.988.

### Identification of *EWSR1*::*VGLL1* fusion and transcriptomic analysis

Total RNA was extracted from FFPE sections using the AllPrep DNA/RNA FFPE Kit (QIAGEN). RNA sequencing (RNA-seq) libraries for the present case were prepared using the TruSeq RNA Exome Kit (Illumina), a capture-based RNA sequencing approach, and sequenced on the Illumina NovaSeq X Plus platform. As a reference cohort, 17 vestibular schwannomas were sequenced on the Illumina NovaSeq 6000 platform using the NEBNext Ultra II Directional RNA Library Prep Kit with rRNA depletion (New England Biolabs, Ipswich, MA, USA).

Fusion gene analysis for both the present case and the 17 vestibular schwannomas reference samples was performed using STAR-Fusion (v1.15.1). The *EWSR1*::*VGLL1* fusion was identified exclusively in the present case and was not detected in any of the vestibular schwannomas. Furthermore, in the present case, the fusion event was validated, and structural breakpoints were characterized using Arriba (v2.4.0), confirming an in-frame *EWSR1*::*VGLL1* fusion. The fusion junction connected *EWSR1* exon 8 to *VGLL1* exon 2 (Fig. 4b).

For transcriptomic analysis, FASTQ files were quantified using Salmon in quasi-mapping mode against the human reference transcriptome (GENCODE v33, GRCh38). Transcript-level abundance estimates were summarized to gene-level counts using the tximport (v1.30.0). Raw counts were normalized using the variance stabilizing transformation (VST) method implemented in DESeq2 (v1.44.0). To evaluate *VGLL1* upregulation in the present case, *VGLL1* expression was compared with that in a reference cohort of 17 conventional vestibular schwannomas analyzed at our institution. To further explore whether *VGLL1* expression was associated with *VGLL1* fusion rather than representing a general feature of intraparenchymal schwannomas, we additionally included RNA-seq data from a non-VGLL-fused intraparenchymal schwannoma harboring a *KPNA4*::*WWTR1* fusion gene [29]. This case was derived from a previously reported non-vestibular schwannoma cohort generated by members of the present author group and was processed using the same expression quantification and normalization pipeline. Because each intraparenchymal schwannoma case consisted of a single sample, relative expression levels were assessed by calculating Z-scores against the distribution of the vestibular schwannoma reference samples. *VGLL1* mRNA was markedly upregulated only in the present *EWSR1*::*VGLL1* fusion-positive intraparenchymal schwannoma, whereas the non-VGLL-fused intraparenchymal schwannoma showed an expression level comparable to that observed in conventional vestibular schwannomas (Fig. 4c).

## Discussion and conclusions

Intraparenchymal schwannomas pose both diagnostic and pathogenetic challenges because of their rarity and their occurrence in locations atypical for tumors of Schwann cell origin. Even in the fifth edition of the World Health Organization Classification of Tumours of the Central Nervous System [18], intraparenchymal schwannomas are not recognized as a distinct entity and are mentioned only under the category of schwannomas. This report describes a case in a 24-year-old woman, contributing to the limited but expanding literature on this rare entity. Since the first report by Gibson et al. in 1966 [8], intraparenchymal schwannomas have been reported throughout the central nervous system (CNS), including the cerebrum, cerebellum, brainstem, and spinal cord [3, 19]. These tumors most commonly occur in young adults, with brainstem lesions being particularly uncommon [10, 15].

Radiologically, intraparenchymal schwannomas often appear as well-circumscribed solid and cystic masses on MRI. They typically show hypointensity on T1WI and hyperintensity on T2WI, with variable contrast enhancement, often avid and homogeneous in the solid components [35]. Calcification, cyst formation, and peritumoral brain edema have also been frequently reported [12, 20, 35]. However, none of these features are sufficiently specific to clearly distinguish intraparenchymal schwannomas from other intra-axial tumors. Therefore, because of their purely intraparenchymal location, it is especially challenging to suspect this tumor preoperatively [12, 19]. The radiological differential diagnosis is broad and includes pilocytic astrocytoma, hemangioblastoma, medulloblastoma, pleomorphic xanthoastrocytoma, ganglioglioma, ependymoma, and metastatic tumors. In the present case, the imaging findings closely resembled those of a pilocytic astrocytoma, and the intraoperative histopathological findings were consistent with a low-grade glioma, exemplifying the difficulty of preoperative diagnosis of intraparenchymal schwannoma.

Histologically, these tumors are characterized by the distinctive biphasic Antoni A and B areas, similar to conventional schwannomas. However, because they develop in areas with no obvious connection to peripheral nerves, it is generally very difficult to make a diagnosis based solely on morphological features. Immunohistochemistry is essential for evaluating Schwann cell differentiation. The immunohistochemical profile generally resembles that of peripheral schwannomas, with S-100 protein and vimentin typically positive and EMA negative [19]. In a histological review of 41 cases by Khoo et al., GFAP was negative in 30 out of 35 cases [12]. Nevertheless, GFAP-positive cases have also been reported, including a single case harboring a *VGLL3* fusion gene [5, 21, 27]. Interestingly, in a recent study of 20 intraparenchymal schwannomas with *VGLL* alterations, all tumors were GFAP-positive. Although this finding may be related to the unique intraparenchymal localization of these tumors, the underlying biological significance remains uncertain. In the present case, which harbored a VGLL alteration, however, GFAP staining was negative.

Regarding prognosis, like the generally benign course of peripheral schwannomas, intraparenchymal schwannomas also tend to show favorable outcomes following complete resection [19]. The recurrence rate after gross total resection has been reported to be 5.3% [15]. Furthermore, VGLL-fused schwannomas showed no recurrence during a median follow-up of 1.8 years, even in cases that underwent subtotal resection [25]. These findings suggest that VGLL-fused intraparenchymal schwannomas follow an indolent clinical course.

Most intraparenchymal schwannomas appear to show a low Ki-67 labeling index [5, 7, 12, 27, 35], similar to conventional peripheral schwannomas. However, the present case demonstrated a relatively elevated Ki-67 labeling index (3.9%). Interestingly, the previous report of schwannomas harboring *VGLL3* fusions has also described relatively elevated Ki-67 labeling index (up to approximately 4%) [21]. However, whether *VGLL* fusion is associated with increased proliferative potential remains unclear. Ishigami et al. reported a case with a high Ki-67 labeling index that showed early postoperative regrowth of residual tumor. This finding suggested that elevated proliferative activity may be associated with a higher risk of early recurrence [10]. Notably, local radiotherapy provided durable tumor control over several years, indicating that adjuvant radiation therapy may be an effective treatment option. In the present case as well, the residual lesion showed early postoperative enlargement but was controlled by stereotactic radiotherapy.

*NF2* mutation is the most frequent genetic alteration in sporadic schwannomas, and its biallelic inactivation is a key mechanism of tumorigenesis. The *NF2* gene product, merlin, functions as a tumor suppressor involved in the regulation of multiple oncogenic signaling pathways, including the Hippo signaling pathway [30]. Recent molecular studies have further revealed additional driver alterations such as *SOX10* mutations and *SH3PXD2A*::*HTRA1* or *TANC1*::*HTRA1* fusions [1, 32, 33]. In addition, other alterations in chromatin regulatory factors and Hippo pathway–related genes, such as *ARID1A*, *ARID1B*, *DDR1*, *LATS1*, and *LATS2*, have been reported in schwannomas [1, 22].

Although several hypotheses regarding the origin of intraparenchymal schwannomas have been proposed, their pathogenesis remains unclear. Molecular characterization of these tumors has historically been limited. Ishigami et al. reported a brainstem schwannoma that lacked genomic alterations commonly associated with schwannomas, including *NF2* mutations and loss of chromosomal 22q [10]. A small number of cases harboring *VGLL1* or *VGLL3* fusion genes have been reported [16, 21]. More recently, these rare tumors have been recognized as a distinct epigenetic cluster, designated as “intraparenchymal VGLL-fused CNS schwannomas” [25]. In this cohort, VGLL family fusions were identified in more than 90% (16/17) of these VGLL-fused schwannomas, with recurrent occurrences of *CHD7*::*VGLL3* and *EWSR1*::*VGLL1* fusions. The presence of these recurrent fusion genes suggests a unique pathogenetic mechanism different from that of *NF2*-driven peripheral schwannomas.

The VGLL family consists of four members, *VGLL1*–*4*, which function as cofactors for TEAD transcription factors through the TDU motif [34]. Functionally, VGLL family proteins act downstream of the Hippo signaling pathway, where they compete with YAP/TAZ for binding to TEAD, thereby modulating gene expression and influencing cell proliferation and tumorigenesis [6, 24]. Interestingly, a brainstem intraparenchymal schwannoma harboring a *YAP1*::*MAML2* fusion in an NF2-wildtype background has also been reported [11].

*EWSR1* fusion genes are well-known oncogenic drivers, particularly in mesenchymal neoplasms, and are characterized by a wide variety of fusion partners. In most EWSR1 fusion proteins, the N-terminal transcriptional activation domain of *EWSR1*, encoded by exons 1–7, is retained and fused to the functional domain of the partner gene, resulting in aberrant transcriptional programs [28]. To date, several cases harboring *EWSR1*::*VGLL1* fusion have been reported. In the mesenchymal neoplasm described by Komatsu et al., the fusion consisted of an in-frame fusion between *EWSR1* exon 9 and *VGLL1* exon 2 [14]. In addition, Kundishora et al. reported a pediatric neuroepithelial neoplasm harboring an in-frame fusion between *EWSR1* exon 9 and *VGLL1* exon 1 [16].

In our case, an in-frame fusion involving *EWSR1* exons 1–8 and *VGLL1* exons 2–5 was identified. *VGLL1* exons 2–5 were retained, suggesting preservation of the functional domains of VGLL1, including the TDU motif located within exon 2, which is essential for TEAD binding [24, 26, 34]. In the reported cases with available breakpoint information, both the transcriptional activation domain of EWSR1 and the TEAD-binding domain of VGLL1 were retained, suggesting that transcriptional activation through the VGLL1–TEAD axis may play an important role in the tumorigenic mechanism of this fusion gene.

Moreover, Schmid et al. also reported that copy number profiling revealed sporadic chromosomal alterations involving chromosomes 3, 8, 19, and 22, without recurrent patterns [25]. In the present case, loss of chromosome 22q was not identified, whereas loss of chromosome 19 was observed. However, previously reported schwannoma-related genetic alterations have not been clearly linked to chromosome 19. Therefore, the biological significance of chromosome 19 loss in this case remains unclear and should be interpreted cautiously.

In intraparenchymal schwannomas with the *EWSR1*::*VGLL1* fusion described by Schmid et al*.*, single-nucleus RNA-seq demonstrated that *VGLL1* expression was elevated in tumor cells compared with non-neoplastic cells, whereas *EWSR1* was not upregulated, suggesting that *VGLL1* overexpression may contribute to tumor development [25]. Interestingly, elevated *VGLL1* expression has been associated with poor prognosis in other cancers, such as ovarian, breast, and gastric cancers [4, 13, 17], indicating that the VGLL1–TEAD axis could represent an important driver of tumor progression.

In the present case, *VGLL1* expression was higher than that observed in typical vestibular schwannomas, whereas the *EWSR1*::*VGLL1* fusion was not detected in any of the vestibular schwannoma reference samples. In addition, the non-VGLL-fused intraparenchymal schwannoma from our previously reported cohort did not show *VGLL1* upregulation. These findings further support the possibility that fusion-driven *VGLL1* activation may contribute to the pathogenesis of intraparenchymal schwannomas harboring *VGLL1* fusions.

Taken together, these findings support the concept that VGLL-fused intraparenchymal schwannomas represent a biologically distinct subgroup separate from conventional schwannomas. The identification of the recurrent *EWSR1*::*VGLL1* fusion and elevated *VGLL1* expression in the present case, together with a distinct DNA methylation profile, provides additional molecular evidence supporting this concept. However, given that non-VGLL fusion events, including *YAP1*::*MAML2* and *KPNA4*::*WWTR1* fusions, have also been reported in intraparenchymal schwannomas, this tumor subset may include molecularly heterogeneous tumors characterized by distinct fusion events.

This study has several limitations. First, as DNA sequencing analysis was not performed, driver alterations reported in peripheral schwannomas (e.g., *NF2*, *SOX10*) could not be fully assessed. Second, DNA methylation and gene expression analyses were performed on a single *VGLL1* fusion-positive intraparenchymal schwannoma case, which restricts statistical evaluation, the reliability of comparisons, and the generalizability of the findings. Nevertheless, observations reported by an independent research group were also confirmed in our case, providing supportive evidence for the biological relevance of *VGLL* alterations.

In conclusion, intraparenchymal schwannoma is a rare but important consideration in the differential diagnosis of well-circumscribed intra-axial CNS tumors. Accurate diagnosis requires careful histopathological and immunohistochemical evaluation, and molecular analysis is playing an increasingly important role. The recent identification of *VGLL1* and *VGLL3* fusion genes, along with distinct DNA methylation profiles, has defined a specific molecular subset.

This study provides additional evidence supporting the pathogenetic relevance of *VGLL* alterations in this tumor subset. In particular, the present case independently confirmed the presence of an *EWSR1*::*VGLL1* fusion together with methylation findings consistent with the recently proposed VGLL-fused intraparenchymal schwannomas. Furthermore, the tumor demonstrated elevated *VGLL1* expression compared with conventional vestibular schwannomas, supporting the potential biological relevance of fusion-driven VGLL1 activation. Further accumulation of integrated molecular and clinicopathological data will be essential to determine whether this subset warrants recognition as a distinct entity within the current CNS tumor classification framework.

## Data Availability

The datasets are available from the corresponding author upon reasonable request.

## Abbreviations

MRI: Magnetic resonance imaging
CT: Computed tomography
T1WI: T1-weighted images
T2WI: T2-weighted images
T2*WI: T2*-weighted images
ADC: Apparent diffusion coefficient
FFPE: Formalin-fixed paraffin-embedded
CNA: Copy number alteration
RNA-seq: RNA sequencing
VST: Variance stabilizing transformation
CNS: Central nervous system
